# The Triglycerides and Glucose Index rather than HOMA-IR is more associated with Hypogonadism in Chinese men

**DOI:** 10.1038/s41598-017-16108-8

**Published:** 2017-11-20

**Authors:** Kun Zhang, Yi Chen, Lijie Liu, Meng Lu, Jing Cheng, Fengbin Gao, Ningjian Wang, Zhoujun Shen, Yingli Lu

**Affiliations:** 1 Institute and Department of Endocrinology and Metabolism, Shanghai Ninth People’s Hospital, Shanghai JiaoTong University School of Medicine, Shanghai, China; 2 Department of Urology, Shanghai Six People’s Hospital, Shanghai JiaoTong University School of Medicine, Shanghai, China; 3 Department of Urology, Huashan Hospital, Fudan University, Shanghai, China

## Abstract

Previous studies have reported that insulin resistant and low testosterone are related. The triglyceride and glucose index (TyG) well mirrors insulin sensitivity. No study investigated the application of TyG in male hypogonadism. We aimed to explore whether TyG was associated with hypogonadism, and also evaluate the ability of TyG compared to HOMA-IR as a possible hypogonadism predictor. A total of 4299 male subjects were enrolled from 22 sites in East China. Hypogonadism was defined as total testosterone <11.3 nmol/L. 695 (16.2%) hypogonadal men had significantly higher TyG index. The prevalence of hypogonadism stepwise increased across increasing TyG quartiles (P < 0.01). TyG was negatively associated with sex hormones and hypogonadism after adjustment for age, current smoking status, hypertension and overweight/obesity (all P for trend <0.01). The full-adjusted odds ratio was 6.1 for the highest quartile compared with the lowest quartile of TyG (95% CI 4.51, 8.25, P < 0.001). On ROC curve analysis, a larger area under the curve was found for TyG (0.71, 95% CI 0.69,0.73) than for HOMA-IR (0.68, 95% CI 0.66,0.70). Thus, the TyG was significantly associated with a higher prevalence of hypogonadism in Chinese men. TyG had a better predictive power for hypogonadism than HOMA-IR.

## Introduction

Male hypogonadism is characterized by reduced serum testosterone concentration, which causes a constellation of clinical signs and symptoms. Currently, the prevalence of hypogonadism among American males aged 45 years or older is estimated to be 38.7%^1^. In Asia, approximately a quarter men has testosterone deficiency and this number is still on the rise^2^. High prevalence of hypogonadism inevitably brings many detrimental impacts on the health and quality of life of men because testosterone deficiency will increase the risk of sexual dysfunction and contribute to presence of comorbid conditions, such as diabetes^3^, obesity^4,5^, cognitive dysfunction^6,7^ and renal failure^8^.

It was well established that insulin resistance (IR) was associated with the increased risk of testosterone deficiency, and testosterone treatment in turn achieved an improvement in insulin signal transduction^9^. The most common index to assess insulin sensitivity is the homeostasis model assessment of IR(HOMA-IR). However, the TyG index, the product of fasting blood glucose (FBG) and triglycerides (TG), is a novel index that has been suggested as a surrogate of insulin resistance in many published literatures^10–12^. Bonora *et al*. reported that correlation coefficients of TyG and HOMA-IR with the euglycemic-glucose clamp were similar, supporting that TyG would be an accessible and reliable tool for clearly reflecting insulin resistance^13^. Another literature from Vasques.*et al*. indicated that TyG index performed better than HOMA to estimate insulin resistance with hyperglycemic clamp in Brazilian subjects^14^. Additionally, the TyG had been demonstrated to be strongly associated with diabetes, obesity, NAFLD and even metabolic syndrome, all of which were risk factors of hypogonadism^15–18^. To the best of our knowledge, no study has investigated the application of TyG in male hypogonadism.

Using data based on the Survey on Prevalence in East China for Metabolic Diseases and Risk Factors (SPECT-China) conducted in 22 sites between 2014 and 2015, we aimed to explore whether TyG was associated with testosterone deficiency, and the ability of TyG compared with HOMA-IR to predict the prevalence of male hypogonadism risk in Chinese men.

## Results

### Characteristics of the study population

The general characteristics of the population are shown in Table 1. Six hundred and ninety-five (16.2%) men were diagnosed with hypogonadism by total T level. As expected, men with hypogonadism had significantly higher levels of TyG, HOMA-IR, fasting insulin, body mass index (BMI), waist circumference, TG, total cholesterol (TC) as well as higher prevalence of hypertension and diabetes (all P for trend <0.001). These subjects also had lower levels of E2, SHBG and LH (all P for trend < 0.001).

**Table 1:** General characteristics of the study population. Continuous variables are presented as the mean (standard.

Characteristic	Men without hypogonadism	Men with hypogonadism	P for trend
N	3604	695	
Age, years	54.03 (13.27)	53.02 (12.26)	0.06
Body mass index, kg/m^2^	24.52 (3.21)	26.48 (3.55)	<0.001
Waist circumference, cm	83.48 (9.43)	88.41 (9.91)	<0.001
Fasting blood glucose, mmol/L	5.62 (1.38)	6.20 (2.02)	<0.001
Fasting Insulin, mmol/L	35.49 (41.22)	55.59 (63.98)	<0.001
HOMA-IR	1.35 (2.71)	2.21 (3.03)	<0.001
TyG	8.74 (0.60)	9.26 (0.75)	<0.001
Triglycerides, mmol/L	1.70 (1.49)	2.96 (3.34)	<0.001
Total cholesterol, mmol/L	5.12 (0.99)	5.25 (1.03)	<0.01
Total T, nmol/L	18.25 (6.37)	9.16 (4.87)	<0.001
E2, nmol/L	112.54 (64.77)	81.53 (50.45)	<0.001
SHBG, nmol/L	50.29 (25.42)	28.78 (15.42)	<0.001
FSH, IU/L	8.90 (7.28)	9.54 (10.31)	0.684
LH, IU/L	5.96 (3.85)	5.39 (4.35)	<0.001
Hypertension, n (%)	50.6	59.6	<0.001
Diabetes, n (%)	13.0	26.6	<0.001
Current smoker, n (%)	49.3	42.6	<0.01

deviation), and categorical variables are expressed as a proportion (%). P for trend was calculated by the Mann-Whitney.

U or Student T test.

Table 2 presents the features of the population according to the TyG quartiles. We observed that the levels of HOMA-IR, fasting insulin, BMI, WC, TG, TC, FBG as well as the prevalence of hypertension and diabetes stepwise increased, while serum total T, E2, SHBG, FSH and LH level stepwise declined with the increasing TyG quartiles (all P for trend < 0.01).

**Table 2:** Characteristics of the study population according to TyG quartiles. Continuous variables are presented as the mean (standard deviation), and categorical variables are expressed as a proportion (%). P for trend was calculated by ANOVA and Chi-square test.

Characteristic	Q1	Q2	Q3	Q4	P for trend
	≤8.35	8.36–8.74	8.75–9.18	≥9.19	
Age, years	47.38 (13.95)	52.98 (12.56)	56.41 (11.86)	56.61 (11.56)	0.23
Body mass index, kg/m^2^	23.17 (3.00)	24.32 (3.20)	25.48 (3.09)	26.40 (3.09)	<0.001
Waist circumference, cm	79.69 (8.83)	82.64 (8.95)	86.10 (9.17)	88.95 (8.98)	<0.001
Fasting blood glucose, mmol/L	5.12 (0.61)	5.37 (0.73)	5.67 (1.13)	6.67 (2.36)	<0.001
Fasting insulin, mmol/L	26.20 (17.99)	32.70 (24.84)	42.67 (40.41)	52.77 (72.48)	<0.001
HOMA-IR	0.87 (0.64)	1.13 (1.02)	1.58 (2.01)	2.37 (4.83)	<0.001
Triglycerides, mmol/L	0.81 (0.16)	1.23 (0.19)	1.76 (0.34)	3.73 (3.07)	<0.001
Total cholesterol, mmol/L	4.76 (0.34)	5.00 (0.96)	5.24 (1.10)	5.60 (1.16)	<0.001
Total T, nmol/L	19.50 (6.37)	17.84 (6.59)	15.87 (5.15)	13.80 (4.87)	<0.001
E2, nmol/L	122.54 (64.77)	112.82 (65.06)	108.81 (61.12)	99.49 (59.55)	<0.001
SHBG, nmol/L	59.31 (28.78)	50.76 (26.00)	43.13 (21.80)	35.30 (17.40)	<0.001
FSH, IU/L	10.26 (10.90)	8.89 (7.00)	9.22 (8.68)	8.07 (5.99)	<0.01
LH, IU/L	6.58 (5.12)	5.83 (3.49)	5.86 (4.11)	5.26 (2.87)	<0.001
Hypertension, n (%)	42.8	47.1	56.3	62.1	<0.001
Diabetes, n (%)	6.1	8.7	13.4	32.4	<0.001
Current smoker, n (%)	55.2	54.7	50.3	47.2	<0.01

**Figure 1:**
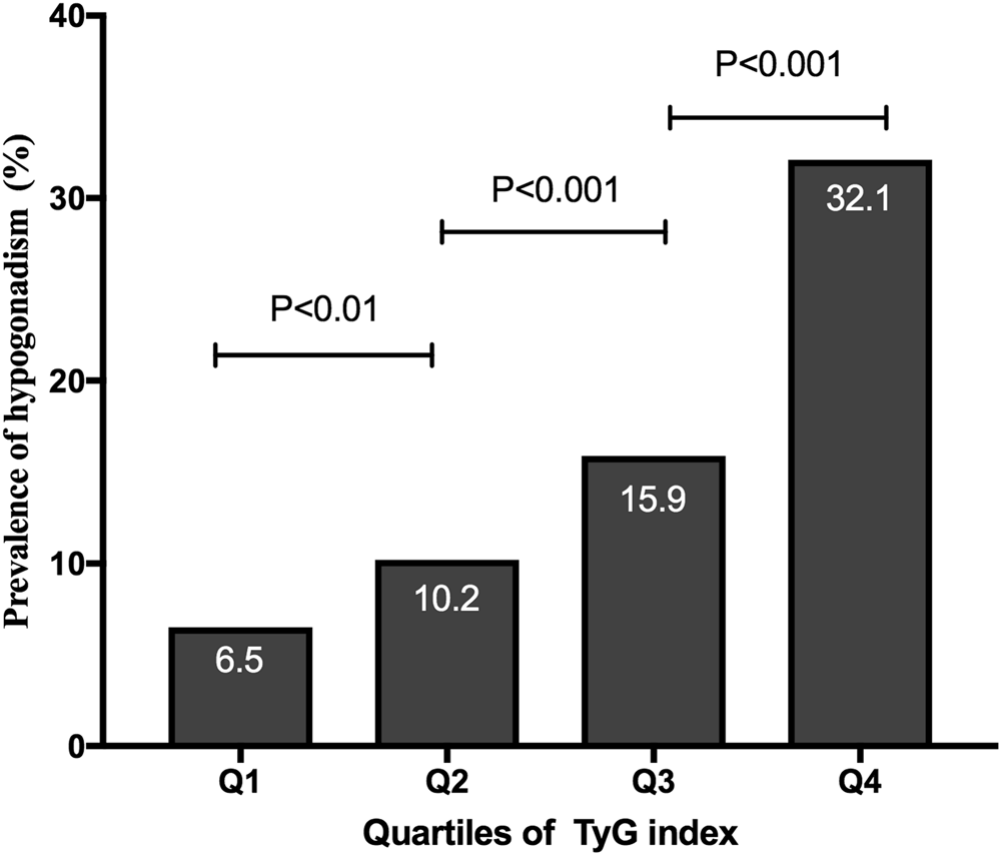
The prevalence of hypogonadism in each quartile of TyG.

Figure 1 presented the prevalence of male hypogonadism in TyG quartile from the lowest to the highest. They were 6.5%, 10.2%, 15.9% and 32.1%, respectively. (all P < 0.01).

### Association of TyG and sex hormones

As described in Table 3, the liner regression results showed that in the model adjusted for age, current smoking status, hypertension and overweight/obesity, the TyG was negatively associated with log-TT, log-E2, log-SHBG, log-FSH and log-LH (all P for < 0.01). Among these hormones, this inverse correlation was much stronger in log-TT (adjusted OR −0.32, 95% CI −0.20, −0.17, P < 0.001) and log-SHBG (adjusted OR −0.30, 95% CI −0.25, −0.21, P < 0.001).

**Table 3:** Association between TyG index and sex hormones. TyG index:

Dependent variables	TyG index (Independent variables)	
	Beta coefficients (95% CI)	P value
log-TT	−0.32 (−0.20, −0.17)	<0.001
log-E2	−0.16 (−0.18, −0.12)	<0.001
log-LH	−0.06 (−0.07, −0.03)	<0.001
log-FSH	−0.04 (−0.06, −0.01)	<0.01
log-SHBG	−0.30 (−0.25, −0.21)	<0.001

Ln[triglyceride(mg/dl) × glucose (mg/ dl)/2]. Values were log-transformed to

approximate a normal distribution. Adjusted for age, current smoking status,

hypertension and overweight/obesity. Liner regression analysis was performed.

### Association of TyG and male hypogonadism

**Figure 2:**
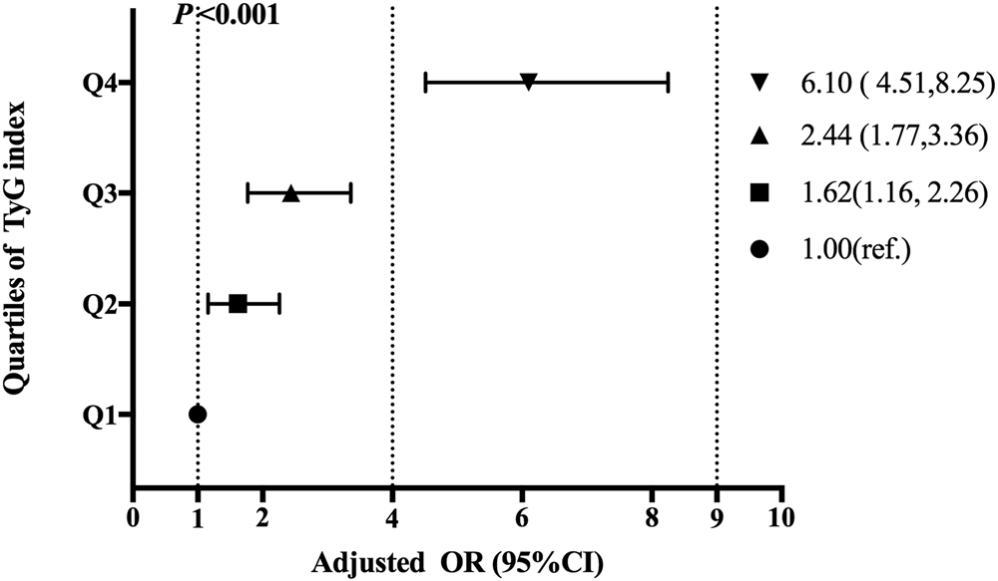
OR of male hypogonadism by TyG index quartile. The data from logistic regression analysis are expressed as odds ratio (95% CI) unless otherwise indicated.

Figure 2 showed the logistical regression results investigating the correlation of TyG with male hypogonadism. The risk of hypogonadism increased across TyG quartiles. Compared with male participants in the lowest quartile (reference), the risk of hypogonadism was 6.1 times higher in those in the highest quartile (OR 6.10, 95% CI 4.51, 8.25) and 2.44 times higher in those in the third quartile (OR 2.44, 95% CI 1.77, 3.36) and 1.62 times higher in those in the second quartile (OR 1.62, 95% CI 1.16, 2.26). ORs were adjusted for age, current smoking status, hypertension and overweight/obesity.

### Predict powers of TyG and HOMA-IR for hypogonadism

**Figure 3:**
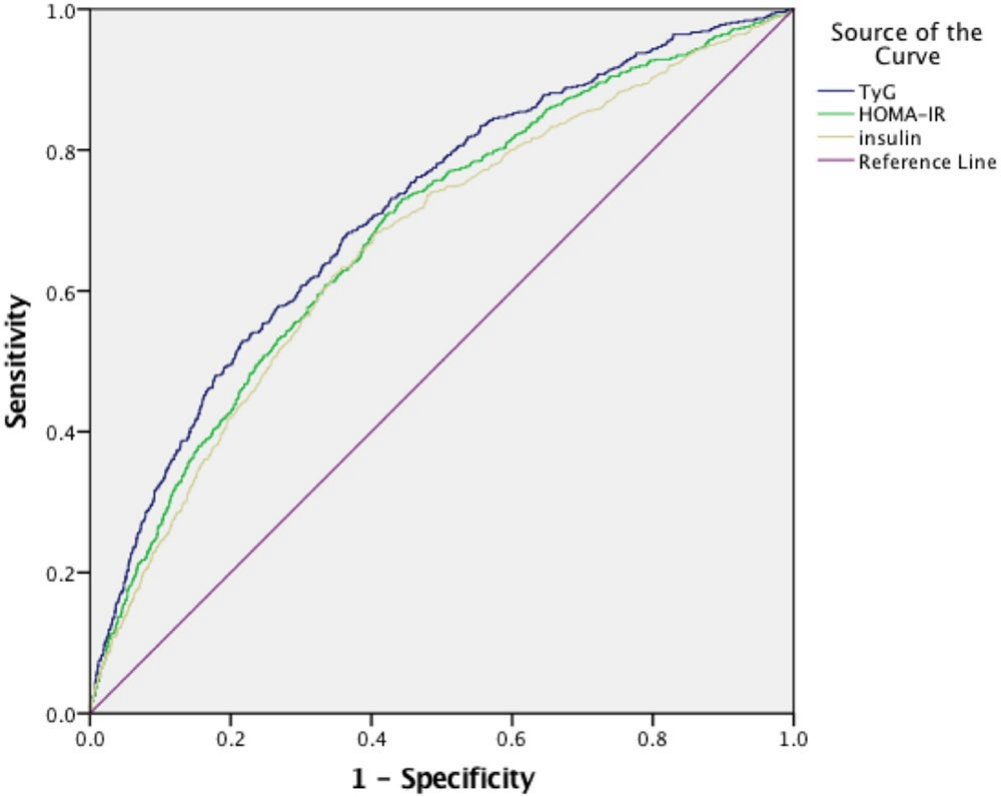
AUROCs for diagnostic accuracy of TyG in comparison to other IR indices. The TyG has the largest area under curves among these indices, suggesting TyG can be a promosing predictive index for male hypogonadism.

The diagnostic value of the indices for predicting hypogonadism was shown in Fig. 3. The AUROC (95%CI) were 0.71(0.69, 0.73) for TyG, 0.68 (0.66, 0.70) for HOMA-IR, and 0.66 (0.64, 0.68) for fasting insulin (Table 4). All the biomarkers were significant predictors for the risk of hypogonadism (all P < 0.001). These results indicated that TyG could effectively differentiate participants with male hypogonadism from those without, and exhibit relative superiority over HOMA-IR and fasting insulin in the diagnostic accuracy. Moreover, when we excluded the subjects being in the use of oral hypoglycemic, lipid-lowing agents and insulin, or with severe hypertriglyceridemia, the ROC values did not appreciably alter compared to the analyses that included these participants (see Supplemental Tables S1 and S2). Additionally, the cut-off point of the TyG index for diagnosis of hypogonadism in Chinese population corresponded to 8.88, with a sensitivity of 0.68 and a specificity of 0.64.

**Table 4:** Comparison of predicting powers between TyG and HOMA-IR for hypogonadism. AUC, area under the curve; ROC, receiver operating characteristic. P value 1: the diagnostic value for ROC, two tail significance. P value 2: the comparisons of AUC between TyG and other IR indices (Z test).

Variable	AUROC(95%CI)	P1 value	P2 value
TyG	0.71 (0.69,0.73)	<0.001	<0.001
HOMA-IR	0.68 (0.66,0.70)	<0.001	<0.001
Insulin	0.66 (0.64,0.68)	<0.001	<0.001

## Discussion

This is the first study to show that the TyG reflecting insulin sensitivity is negatively associated with lower total T, E2, LH, FSH and SHBG concentrations after adjustment for potential confounders (age, current smoking status, hypertension and overweight/obesity). The full adjusted OR of hypogonadism in the highest quartile of TyG grow 6.1 folds compared with that in the lowest quartile. We also found that TyG could recognize male hypogonadism accurately with a AUC of 0.71 (0.69, 0.73), exhibiting obvious superiority over HOMA-IR and fasting insulin.

The TyG was regarded as a new surrogate of insulin resistant, first proposed by Guerrero-Romero *et al*. in 2008^10^. Recent researches further indicated that TyG also correlated with cardiovascular disease^19^, obesity, NAFLD and metabolic syndrome, all of which were critical risk factors of hypogonadism. However, no study investigated the application of TyG index in hypogonadism. Given HOMA-IR and fasting insulin had been reported in literatures as valuable biomarkers of low testosterone^20,21^, we compared the predictive ability of TyG and these two indices for incidence hypogonadism. All the parameters in AUROC analysis were significant predictors for future risk of male hypogonadism. TyG had larger AUROC than these known indices. Thus the TyG could be used as a surrogate of hypogonadism and we provided new evidence to application of TyG.

However, it should be noted that AUROC for the TyG index was about 0.71, which means only approximately 70% individuals with low testosterone could be accurately identified. Meanwhile, some clinical pathological conditions, such as dyslipidemia, metabolic syndrome, might influence the values of TyG index in its application for the screening of hypogonadism.

Insulin resistant and male hypogonadism are closely linked. In our study, the levels of fasting insulin, HOMA-IR and TyG index were significantly higher in the hypogonadic men than those in the eugonadic men. These results were in accordance to the previous study of an inverse correlation between testosterone level and insulin resistance^22^. Meanwhile, our results also showed that insulin resistant reflected by TyG was significantly associated with sex hormones and the risk of male hypogonadism. Although obtained many findings, how IR influence hypogonadism is still unknown. Salvi *et al*. reported that insulin action and insulin sensitivity in the brain were indispensable for the maintenance of the functional integrity of the hypothalamic-pituitary-gonadal axis^23^. The selective deletion of the insulin receptor from neurons in mice resulted in a significant decrease in LH and testosterone concentrations, and incubation of hypothalamic neurons with insulin led to the enhancement of secretion of GnRH^24,25^. Thus we supposed that hypothalamic dysfunction with a reduced gonadotropin-rereleasing hormone level was responsible for the hypogonadism condition, because levels of E2, FSH and LH in hypogonadic men were lower, which was more obvious when TyG quartiles were took into account.

Another unexpected finding was that a significant inverse relationship existed between SHBG concentration and TyG independent of important confounders. Extensive epidemiological studies indicated that lower circulating concentration of SHBG was related to higher insulin resistance^26–28^. Serum SHBG level could mirror the degree of inflammation in many metabolic disorders, including insulin resistant and diabetes^29^. Another study found that insulin per se could directly inhibit SHBG secretion from hepatoma cells *in vitro* ^30^. This further indicated IR reflected by TyG contributed to the regulation of sex hormones.

There are many strengths in our study. First, concerning the novelty, this is the first time to point out a strong correlation between the TyG and testosterone deficiency in general Chinese men, providing new application in this field. Second, regarding the clinical application, the TyG can be served as a more promising tool for recognizing subjects with hypogonadism than HOMA-IR, especially among subjects of low income living in disadvantaged socioeconomic environments in which there is no availability for measurement of testosterone. Third, all the questionnaires and measurements were performed by the same trained personnel and laboratory center, thus providing strong quality control.

This study also had some limitations. First, we did not measure serum albumin concentration, so calculated free T level could not be obtained. However, diagnosis of hypogonadism based on total T had been accepted and applied in extensive epidemiological studies^31–33^. Second, we did not define androgen deficiency by incorporation of signs/symptoms and testosterone level. In fact, most prevalence estimates were determined by low testosterone level alone mainly because men with low testosterone level may not exhibit clinically classical symptomatology. Third, given the effect of ethnicity on the variability of triglyceride level, further study is needed to assess the TyG index in different populations.

In conclusion, our results showed that TyG index was significantly associated with hypogonadism and exhibited obvious superiority over HOMA-IR in predicting low testosterone in general Chinese men, suggesting that it could be a promising tool to identify subjects with hypogonadism.

## Methods

### Study population

SPECT-China is a population-based cross-sectional investigation to assess the prevalence of metabolic disease and risk factors in East China. Registration number is ChiCTR-ECS-14005052(www.chictr.org.cn). This investigation was approved by the Ethics Committee of Shanghai Ninth People’s Hospital, Shanghai JiaoTong University School of Medicine, and all study subjects signed an informed consent before data collection. Study procedures were in accordance with the ethical standards of the responsible committee on human experimentation (institutional and national) and with the Helsinki Declaration of 1975, as revised in 2008. This survey was conducted from 2014 January to 2015 December. A stratified cluster sampling method was performed and the detail sampling process had been reported in previous published studies^34,35^. People aged over 18 years who had lived in their current residence for 6 months or longer were invited to participate in the study. Those with acute illness, serious communication disorders, or who were unwilling to cooperate were excluded. 10,441 adult subjects were recruited from 22 sites in Shanghai, Zhejiang, Jiangsu, Jiangxi and Anhui Province. Among them, there were 4309 men who never received testosterone supplement treatment. We further excluded participants who were missing results of triglyceride (TG, n = 1) or fasting blood glucose (FBG, n = 2) or total testosterone (total T, n = 7). At last, a total of 4299 male subjects were enrolled in the study.

### Anthropometric and laboratory measurements

Questionnaire was used to collect data of study participants about demographic characteristics, medical history and lifestyle risk factors. This process was completed by the same trained physicians and students at every survey site. Height and body weight were measured with subjects wearing light clothing and no shoes. BMI was calculated by weight (kg)/height(m^2^). WC was measured midway between the inferior border of the last rib and the crest of the ilium at the end of expiration. Blood pressure was measured with sphygmomanometer according to standard technique^36^. Smoking was defined as having smoked at least 100 cigarettes in one’s lifetime and currently smoking cigarettes.

Venous blood samples were drawn from participants after an overnight fast of 8 h or longer. All the biochemical measurements were completed in the central laboratory certified by the College of American Pathologists. The total T, estradiol (E2), follicle-stimulating hormone (FSH) and luteinizing hormone (LH) were measured by the immulite 2000 platform chemiluminescence immunoassays (Siemens, Germany) and SHBG by electrochemiluminescence (Roch Cobas E601, Switzerland). Fasting plasma glucose (FBG), TG, high density lipoprotein (HDL), low density lipoprotein (LDL) and total cholesterol (TC) were tested by BECKMAN COULTER AU 680 (Germany). HbA1c was measured using high-performance liquid chromatography (MQ-2000PT, China).

### Definition and calculation

TyG index was calculated as the logarithm (Ln) of (fasting blood glucose (mg/dL) × TG(mg/dL)/2)^37^. Hypogonadism was defined as total T less than 11.3 nmol/L in men^31^.

Diabetes was defined as a fasting plasma glucose of 7 mmol/L or higher, HbA1c of 6.5% or higher, or a previous diagnosis of type 2 diabetes. Hypertension was defined as systolic blood pressure ≥ 140 mmHg, diastolic blood pressure ≥ 90 mmHg, current use of antihypertensive drug or self-reported history of hypertension. Overweight/obesity was defined by body mass index of at least 25 kg/m^2^. HOMA-IR was estimated by the formula fasting insulin (U/mL) × fasting glucose (mmol/L)/22.5^38^.

### Statistical analysis

The IBM SPSS software, Version 23 (IBM Corporation, Armonk, NY, USA) was used for statistical analyses. Continuous variables were presented as the mean (standard deviation), and categorical variables were expressed as a proportion (%). General characteristics were compared by the Mann-Whitney U or Student T test for continuous variables and Chi-square test for categorical variables. The TyG index was recoded into quartiles and the prevalence of hypogonadism was calculated in each quartile of TyG. We used linear regression analyses to investigate the association of sex hormones with TyG index adjusted for age, current smoking status, hypertension and overweight/obesity. TG and FBG were not adjusted because they were included in the formula of TyG. Total T, E2, LH, FSH and SHBG were log-transformed because of their high skew. In order to explore the relationship between TyG and the risk of hypogonadism, logistic regression was used and the lowest quartile served as the reference. Data were expressed as odds ratio (OR) and 95% confidence interval (CI). Adjusted factors were the same as those in linear regression.

The abilities of TyG index, HOMA-IR and fasting insulin to predict the prevalence of hypogonadism were investigated with receiver operating characteristic (ROC) curves and their respective areas under the curve. Differences between AUCs were compared by Z tests. All statistical analyses were two-sided. *P* < 0.05 was considered statistically significant.

### Data Availability

The datasets generated during or analysed during the current study are available from the corresponding author on reasonable request.

## Electronic supplementary material

Supplementary information
