# Supplementary material for: The Triglycerides and Glucose Index rather than HOMA-IR is more associated with Hypogonadism in Chinese men
Source: Sci Rep. 2017 Nov 20;7:15874. doi: 10.1038/s41598-017-16108-8 (PMC5696537; doi:10.1038/s41598-017-16108-8)
Supplement: Supplementary file 1 — Supplementary information [file 41598_2017_16108_MOESM1_ESM.doc]

**The Triglycerides and Glucose Index rather than HOMA-IR is more associated with Hypogonadism in Chinese men**

Kun Zhang^1#^, Yi Chen^1#^, Lijie Liu^2#^, Meng Lu^1^, Jing Cheng^1^, Fengbin Gao^3^, Ningjian Wang^1^, Zhoujun Shen ^3^*, Yingli Lu^1^*

^1^Institute and Department of Endocrinology and Metabolism (K.Z., Y.C., M.L., J.C., N.W., Y.L.), Shanghai Ninth People’s Hospital, Shanghai JiaoTong University School of Medicine, Shanghai, China; ^2^ Department of Urology (L.L), Shanghai Six People’s Hospital, Shanghai JiaoTong University School of Medicine, Shanghai, China;^3^ Department of Urology (P.G., Z.S.), Huashan Hospital, Fudan University, Shanghai, China

^#^K.Z., Y.C., and L.L. contributed equally to this work.

***Corresponding Author:** Yingli Lu, MD & PhD.

Institute and Department of Endocrinology and Metabolism, Shanghai Ninth People’s Hospital, Shanghai Jiao Tong University School of Medicine, Shanghai, China

No.639 Zhizaoju Road, Shanghai, 200011

Phone: +86-021-23271699-5760; Fax number: +86-21-23271699-5760;

E-mail: luyingli2008@126.com

**^#^Co-corresponding Author**: Zhoujun Shen, MD & Ph.D.

Department of urology, Huashan Hospital, Fudan University, Shanghai, China, 200040. Telephone Number: 86-21-52889999; Fax number: +86-21-52889999;

E-mail: [shenzj68@sina.cn](mailto:shenzj68@sina.cn)

| Variable | AUROC(95%CI) | P1 value | P2 value |
| --- | --- | --- | --- |
| TyG | 0.71 (0.69,0.73) | <0.001 |  |
| HOMA-IR | 0.68 (0.65,0.70) | <0.001 | <0.001 |
| Insulin | 0.66 (0.64,0.69) | <0.001 | <0.001 |

**Supplemental Table S1 Comparison of predicting powers between TyG and HOMA-IR for hypogonadism.** Exclusion of subjects being in the use of oral hypoglycemic and lipid-lowering agents and insulin. P value 1: the diagnostic value for ROC, two tail significance. P value 2: the comparisons of AUC between TyG and other IR indices (Z test).

| Variable | AUROC(95%CI) | P1 value | P2 value |
| --- | --- | --- | --- |
| TyG | 0.71 (0.69,0.73) | <0.001 |  |
| HOMA-IR | 0.69 (0.66,0.71) | <0.001 | <0.001 |
| Insulin | 0.67 (0.65,0.70) | <0.001 | <0.001 |

**Supplemental Table S2 Comparison of predicting powers between TyG and HOMA-IR for hypogonadism.** Additional exclusion of subjects with severe hypertriglyceridemia. P value 1: the diagnostic value for ROC, two tail significance. P value 2: the comparisons of AUC between TyG and other IR indices (Z test).
